# SPOTLIGHTING THE EXPERIENCES OF THE HOME CARE WORKFORCE CARING FOR OLDER ADULTS LIVING WITH DEMENTIA

**DOI:** 10.1093/geroni/igae098.1230

**Published:** 2024-12-31

**Authors:** Chanee Fabius, Joanne Spetz

**Affiliations:** Chair; Discussant

## Abstract

Medicaid Home and community-based services (HCBS) and home-based clinical care (i.e., Medicare home health care) aim to support independence and well-being in the community, especially for older adults living with disability and complex health conditions, such as dementia. Many older adults receive both Medicaid HBCS and home-based clinical care, often concurrently, with services coordinated and delivered by paid and unpaid care partners including care managers, home care workers, and family caregivers. Amid concerns related to workforce shortages, shifting payment policies, and the rising complexity of those aging in place, there have been recent calls to better support the workforce providing care in the home. Responding to these calls will require additional knowledge and understanding of the experiences of workforce members as well as the older adults they serve. This symposium presents four unique studies that describe: 1) experiences of care managers (“supports planners” in Maryland) serving older adults with and without dementia in Medicaid HCBS in Maryland; 2) unmet caregiving needs in a national sample of Medicare home health patients living with dementia; 3) Medicare home health clinician perspectives on supporting dementia caregivers through health information technology, and 4) home care worker attitudes about delivering person-centered care for persons living with dementia. The findings presented reveal current areas of challenge and inform the development of innovative strategies to better support the range of paid and unpaid caregivers caring for older adults living with dementia in the community.

